# The influence of family incivility on cyberbullying perpetration: A moderated mediation model

**DOI:** 10.3389/fpsyg.2022.975335

**Published:** 2022-08-25

**Authors:** Jing Gao, Fang Liu, Jianjun Xu, Jinyu Wang, Jiaqi Mou, Lingchao Wang

**Affiliations:** ^1^International College, Ningbo University, Ningbo, China; ^2^College of Teacher Education, Ningbo University, Ningbo, China; ^3^United Faculty of China and French, Ningbo University, Ningbo, China; ^4^College of Education, Zhejiang University, Hangzhou, China

**Keywords:** family incivility, cyberbullying perpetration, negative emotions, neuroticism, the frustration-aggression theory

## Abstract

Previous research has extended the stress literature by exploring the relationship between family incivility and cyberbullying perpetration, yet relatively less attention has been paid to underlying psychological mechanisms between that relationship among university students. According to the Frustration-Aggression Theory, this study examined the relationships of family incivility, cyberbullying perpetration, negative emotions and neuroticism among Chinese university students. Data were collected from 814 university students (females, *N* = 423; Mage = 19.96 years, SD = 3.09). The results examined the mechanism through which family incivility was significantly related to cyberbullying perpetration through the mediation of negative emotions, suggesting a strong link of stressful life events to online aggression. In addition, high levels of neuroticism moderated the relationship between family incivility and cyberbullying perpetration, as well as that between family incivility and negative emotions. The study revealed the chronic and potential impact of family incivility, underlined the interaction between stressful life events and online aggression, and put forward the intervention strategies of cyberbullying among university students.

## Introduction

Family interaction plays an important role in the development of individual social emotion and cognition (Bronfenbrenner, 1986). Recently, family incivility, a new form of negative family interactions, has attached widespread attention. Family incivility is low-intensity deviant interpersonal behaviors that undermine mutual respect in the family, such as excluding family members from social activities and doubting the judgment of family members (Lim and Tai, 2014; Bai et al., 2016). It is easily ignored since its intensity is low and its consequence is not as immediate as family abuse or violence (Cortina and Magley, 2009), for which the negative family interaction is difficultly restrained and can repeatedly inflict the victims (Bai et al., 2020). Moreover, due to its ambiguity in purposes, the victims usually deem it as unintentional, tolerable, and acquiescent, leading to its long-term existence in our daily life (Sliter et al., 2011).

Previous studies examined the associations between family incivility and adults’ work performance (Naeem et al., 2020; Ren et al., 2021). Less is known, however, about the influence of family incivility on students. A study from China (Bai et al., 2020) indicated that family incivility was positively associated with cyberbullying perpetration among Chinese middle school students. An empirical study found that family incivility had a negative effect on university students’ work engagement in India (Gopalan et al., 2021). However, to our knowledge, no research has been conducted on the influence of family incivility on cyberbullying perpetration among university students. Cyberbullying perpetration is defined as willful and repeated harm of an individual or a group inflicted by computers, cell phones, and other electronic devices (Hinduja and Patchin, 2008; Campbell et al., 2013). This new-form aggression with anonymity, concealment, high dissemination, can result in a variety of negative consequences, for instance, anxiety, depression, and suicidal ideation (Patchin and Hinduja, 2011; Kowalski et al., 2012).

Recently, the booming of Internet and social media has intensified cyberbullying in China (Chu et al., 2021). Early in 2018, Li and his colleagues found that one in three Chinese adults has experienced cyberbullying and one in two Chinese minors has experienced cyberbullying (Li et al., 2018). However, university students as the major net-citizens are more likely involved in cyberbullying than middle school students. In addition, previous studies have shown that family interactions are significantly related to the development of university students’ cognition and behaviors (Wright et al., 2020; Smith and D’Aniello, 2021). Negative family interactions are positively associated problem behaviors among university students (Fortesa and Ajete, 2014). Therefore, it is necessary to investigate how family incivility influences university students’ cyberbullying perpetration, theoretically and empirically, to restrain the prevalence of cyberbullying from the perspective of the frustration-aggression theory.

This study has three-fold contributions. Firstly, although previous studies have been devoted to the influence of family incivility on adolescents’ cyberbullying perpetration (Bai et al., 2020), it is unclear how family incivility might be related to cyberbullying perpetration among university students. To explore the negative effects of family incivility on university students, we establish a moderated mediation model based on the frustration-aggression theory, central to which is negative emotions, while neuroticism moderates the relationship between family incivility and negative emotions as well as that between family incivility and cyberbullying perpetration. Secondly, the empirical findings support the frustration-aggression theory by demonstrating the mechanism through which frustration, such as family incivility, is associated with aggressive behaviors, such as cyberbullying perpetration, and extend the existing theory by observing the interplay between online and offline behaviors. Thirdly, through the chronic and low-intensity negative family interaction, this paper throws new light on the mechanism and intervention of cyberbullying in universities.

## Investigating the relationship between family incivility and university students’ cyberbullying perpetration

The frustration-aggression theory states that frustration can affect the inclination to act aggressively (Berkowitz, 1988). Individuals experiencing more frustration are more likely to perpetrate aggression in the future. However, the anticipation of punishment can influence their choices of the target, as they expect to escape from the repercussions of their aggression and keep themselves from being inflicted (Berkowitz, 1989). In face of parental authority, frustrated individuals may give up fighting back while tending to perpetrate some covert forms of aggression (Bai et al., 2020). The Internet provides a virtual and anonymous space for them to release their negative emotions (Pabian and Vandebosch, 2014), where they can better modify their representation of themselves (Valkenburg and Peter, 2011) and conceal their real identity, to reduce their possibility of being negatively evaluated or retaliatorily attacked by others (Bane et al., 2010). Family incivility as a frustration possibly results in less punitive aggressive acts as well, such as cyberbullying perpetration. Many studies have also suggested that negative family interactions were significantly related to cyberbullying perpetration (Low and Espelage, 2013; Barlett and Fennel, 2016; Lee and Kang, 2019; Romero-Abrio et al., 2019). Barlett and Fennel (2016) found that parental neglect was positively associated with individual cyberbullying perpetration. A cross-sectional study (Romero-Abrio et al., 2019) also found that problematic family interaction was directly associated with online aggressive acts among adolescents. A study of 423 Korean middle school students indicated that high levels of cyberbullying perpetration was significantly associated with low levels of parent-adolescent relationship quality and high levels of parental control (Lee and Kang, 2019). Longitudinal research also found that high level of parental monitoring could significantly predict increasing cyberbullying perpetration after one and a half years (Low and Espelage, 2013). Accordingly, the following hypothesis is proposed:

**H1:** Family incivility will be positively correlated with cyberbullying perpetration among university students.

### Negative emotions as a mediator

Negative emotions are fundamentally a subjective experience of unpleasant or depressed mood in the past week, including various annoying emotional states, e.g., depression, anxiety, and fear (Watson et al., 1988), which may increase individuals’ tendency to bullying or self-injury (Agnew, 1992). Thus, family incivility as a stressful life event may cause the person’s cyberbullying perpetration and increase their intention to perpetrate cyberbullying through negative emotions as well. Empirical studies found that depression, anxiety, and stress were the most common mental illnesses among university students worldwide (Smith et al., 2017; Paudel et al., 2020). The frustration-aggression theory (Berkowitz, 1989) argues that individuals with stressful life events would first produce negative emotions, and then develop an instigation to aggression. Family incivility as a stressful life event can lead to negative emotions. Previous studies have found that family incivility significantly predicated individual emotional consumption (Hassan et al., 2019) and negative emotions (Sarwar et al., 2019). A cross-sectional study found that family incivility, as a subtle and chronic stressful life event among family members resulted in individual psychological distress (Lim and Tai, 2014). A study of 3030 Chinese high school students found that family neglect, rejection, and suspicion made individuals feel hopeless about the future (Bai et al., 2020). A longitudinal study indicated that family incivility lowered employees’ job satisfaction by depleting their psychological resources and causing their stress (Maria et al., 2021).

In addition, Berkowitz (1983) emphasized the role of negative emotions in the frustration-aggression process, arguing that the negative emotions reflected the strength of frustration-produced instigation to aggression. Psychological discomfort or depression activates other negative memories and feelings, thereby promoting individual inclination to aggression (Berkowitz and Heimer, 1989). In other words, family incivility, that is, frustration, may lead to individual negative emotions, which in turn triggers or reinforces their aggressive tendencies, making them more likely to perpetrate aggression for alleviating or getting rid of the negative effects from chronic negative family interactions, such as neglect, contempt, rejection. A longitudinal study found a significant positive correlation between family incivility and bank employees’ counterproductive work behaviors in Pakistan, with psychological distress mediating the direct relationship (Hameed et al., 2017). Another longitudinal study found that depression and anxiety predicted cyberbullying perpetration over time (Laura et al., 2020). A six-year longitudinal study indicated that negative mental factors positively predicted cyberbullying perpetration (You and Lim, 2016). Several studies have also confirmed a significant correlation between negative emotions and cyberbullying perpetration (Balta et al., 2020; Schodt et al., 2021). Accordingly, the following hypothesis is proposed:

**H2:** Negative emotion will mediate the direct relationship between family incivility and cyberbullying perpetration among university students. Specifically, family incivility increases university students’ negative emotions, leading to cyberbullying perpetration.

### Neuroticism as a moderator

Personality is a relatively stable individual trait, which has a long-term impact on individual behavioral style (Back et al., 2009). The Five-Factor Model of personality holds that personality has five basic dimensions: Extraversion, Agreeableness, Conscientiousness, Openness, and Neuroticism (Koivisto et al., 2021). Different personality traits have different influences on individual observation and interaction with environmental stressors (Bai et al., 2020). Neuroticism represents individual differences in the tendency to experience distress (Mccrae and Costa, 1987) and negative mental health outcomes (Anglim et al., 2020), because of which it is widely studied in stress research (Hill and Kemp-Wheeler, 1986; Mineka et al., 2020). Highly neurotic individuals frequently have high levels of anxiety, depression, anger, and guilt, as well as an aggravating somatization tendency of psychological problems, leading to individual cognitive and behavioral differences (Costa and Mccrae, 1992). Those with high levels of neuroticism are more likely to experience stressful and negative events in reality (Miceli et al., 2021).

Previous studies have considered personality as a moderator that influences the association between stressful life events and negative emotions. A cross-sectional study found highly neurotic individuals were more vulnerable to depression in face of stressful life events (Roberts and Kendler, 1999). A case study with 83 survey participants also indicated that individuals with high levels of neuroticism were more vulnerable to external environment, unstable in affection, and sensible to various stimuli, which made them more prone to depression and anxiety (Johan et al., 2002). A meta-analysis study under an organizing framework of the big-five model found that highly neurotic individuals tended to exhibit poor adjustment and were prone to negative emotional states, including nervousness, anxiety, moodiness, and worry (Judge et al., 2002). A one-year longitudinal study demonstrated that highly neurotic individuals were more likely to embrace negative automatic thoughts while suffering some frustrations or failures, leading to their negative emotions (Amy et al., 2009). Another two-year longitudinal study indicated that highly neurotic individuals were more likely to have high levels of depressive symptoms because of stressful life events (Loey et al., 2014). As mentioned above, family incivility is a chronic, imperceptible but influential stressful life event. Therefore, neurotics at a high level appear more sensitive to negative family interactions, such as neglect, exclusion, and contempt by family members, and thereby produce more negative emotions. Accordingly, the following hypothesis is proposed:

**H3:** Neuroticism will moderate the relationship between family incivility and negative emotions, so that the positive correlation between family incivility and negative emotions is stronger for highly neurotic university students, and vice versa.

Berkowitz (2003) argued that aggression was not always a consequence of frustration since the frustration-aggression process was related to more cognitive factors, e.g., personality, understanding of frustration, mentality in the face of frustration and ability to bear frustration. Personality played an indispensable part in the theoretical construct of frustration-aggression model (Berkowitz, 1989). Neuroticism, a typical personality can also affect individuals’ cognitive processing, thereby influencing their aggressive acts (Olver and Mooradian, 2003). Individuals with a higher level of neuroticism were more prone to emotional orientation instead of problematical orientation in selecting coping strategies, making them harder to deal with stressors and consequently adopt negative coping strategies (Horner, 1996), e.g., cyberbullying perpetration. Taylor and Kluemper (2012) found that neurotics at a high level perceived more incivility in workplaces and behaved more aggressive during their work, while neurotics at a low level did not. A prior study found that neuroticism played a moderating role in the influence of stressful life events on aggression (Sun et al., 2016). Accordingly, the following hypothesis is proposed:

**H4:** Neuroticism will moderate the relationship between family incivility and cyberbullying perpetration, so that the positive correlation between family incivility and cyberbullying perpetration is stronger for highly neurotic university students, and vice versa.

**FIGURE 1:**
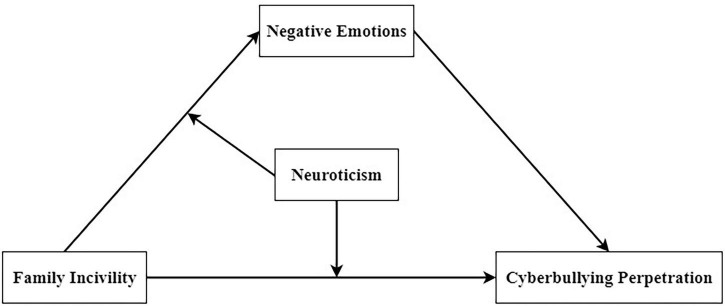
The moderate mediating model.

Taken together, the whole research model is presented in Figure 1.

## Materials and methods

### Participants and procedures

A total of 814 participants of this study were recruited from a university in Zhejiang Province, China. They are aged 17–26 years old [mean(M) ± standard deviation (SD) = 19.96 ± 3.090], with 391 (48%) being males and 423 (52%) being females. Among the participants, 403 (49.5%) were freshmen, 198 (24.3%) were sophomores, 92(11.3%) were juniors and 121(14.9%) were seniors. Average monthly household income ranges from less than 2000 yuan to more than 10000 yuan (10.3%, less than 2000 yuan; 23.3%, 2001–5,000 yuan; 34.8%, 5001–10000 yuan; 31.6%, more than 10000 yuan). Average daily smartphone usage time ranges from 1 h to more than 9 h (12.5%, 1–3 h; 46.1%, 3–6 h; 28.1%, 6–9 h; 13.3%, more than 9 h).

### Measures

#### Family incivility

The family incivility scale was originated from the Workplace Incivility scale (Cortina et al., 2001) modified by Lim and Tai (2014). The scale measured incivility experienced from family members, comprising of six items (e.g., “Ignored or excluded you from social activities?”), which were rated by participants on a five-point scale (1 = not at all and 5 = most of the times). In the present study, the Cronbach’s alph coefficient for the family incivility is 0.926.

#### Negative emotions

The Chinese version of Depression Anxiety and Stress Scale 21 (DASS-21) (Gong et al., 2010) was used to measure the level of negative emotions in half a year and the initial version was developed by Lovibond and Lovibond (1995). The Dass-21 scale with 21 items includes three subscales (i.e., depression, anxiety, and stress with 7 items, respectively) rated on 4-point Likert scale ranging from 0 (“did not apply at all”) to 3 (apply to me very much), with higher scores representing higher levels of negative emotions. Many studies have employed this scale to assess the frequency and severity of three negative emotional states (i.e., depression, anxiety, and stress) among university students (Ng et al., 2010; Aoyama et al., 2011; Chong et al., 2017), including Chinese university students (Xu et al., 2013; Yang et al., 2020), with documented evidence of reliability and validity (Antony et al., 1998; Holfeld and Baitz, 2020). In the present study, the Cronbach’s alph coefficient for the Chinese DASS-21 is 0.954.

#### Cyberbullying perpetration

The Chinese version of Cyberbullying Scale (CVCS) (Xu, 2015) was used in this research, which integrated the scales developed by Olweus (1993); Zhang and Wu (1999), as well as Cretin et al. (2011). CVCS is composed of 12 items (e.g., “Rumoring on the Internet”) that measures the level of cyberbullying perpetration in direct and indirect ways. Participants responded on a 5 – point scale ranging from never (1) to always (5), with higher scores representing higher levels of cyberbullying perpetration. In the present study, the Cronbach’s alph coefficient for the CVCS is 0.943.

#### Neuroticism

The Chinese Big Five Personality Inventory Brief Version (CBF-PI-B) (Wang et al., 2011) was used in this research and initial version was developed by John et al. (1991). The CBF-PI-B is a 40-item scale consisting of 5 personality factors (i.e., Extroversion, Conscientiousness, Neuroticism, Openness, and Agreeableness) and it is rated on 6-point Likert scale ranging from 1 (strongly disagree) to 6 (strongly agree), measuring personality traits as defined by the Five Factors Model (Costa and Mccrae, 1992). For the present study, we analyzed only data from the Neuroticism subscale with 8 items (e.g., “I am relatively stable from an emotional point of view”). We computed the total score by averaging participants’ scores for each of the items of the scale, with higher scores representing higher levels of neuroticism. In the present study, the Cronbach’s alph coefficient for the CBF-PI-B is 0.851.

#### Covariates

The variables of participants’ age, gender, average monthly household income, and average daily smartphone usage time were controlled for, as former studies showed that they might affect negative emotions and cyberbullying significantly (Deb and Walsh, 2012; Anat, 2014; Kim, 2015; Peng et al., 2021).

### Data analysis

Firstly, we calculated descriptive statistics and correlations matrix. To facilitate result interpretation and avoid the multicollinearity problem (Aiken and West, 1991), all the data were standardized except for the dependent variable. Secondly, we used PROCESS macro (Model 4) developed by Hayes (2013) to test the mediation effect of negative problems. Thirdly, we conducted PROCESS macro (Model 8) developed by Hayes (2013) to examine whether neuroticism moderated this mediation process. Additionally, to investigate the significance of indirect effects, we drew on the bootstrapping method (Hayes and Scharkow, 2013), which produces 95% bias-corrected confidence intervals from 5000 resamples of the data. The effects are significant when the confidence intervals exclude zero.

## Results

### Statistical description

As this study aimed at exploring whether negative emotions would mediate the association between family incivility and cyberbullying perpetration and whether this mediation effect would be moderated by neuroticism, the analysis included the following three steps.

### Preliminary analyses

The means, standard deviations, and correlation coefficients for all variables of the current study are displayed in Table 1. The results indicated that the relationship between all variables were statistically significant, and family incivility was positively related to cyberbullying perpetration. Therefore, H1 received support.

**TABLE 1:** Descriptive statistics and correlations for all variables.

	*M*	*SD*	1	2	3	4	5	6	7	8	9
1 Gender	1.52	0.5	1								
2 Age	19.96	3.09	−0.22**	1							
3 AMHI	2.88	0.97	0.10**	−0.09**	1						
4 ADSUT	2.42	0.87	0.12**	0.02	0.14**	1					
5 Grade	1.92	1.09	–0.15	0.61**	−0.09**	–0.00	1				
6 NE	33.9	11.35	−0.13**	0.19**	−0.13**	0.12**	0.09*	1			
7 CP	15.53	6.47	−0.17**	0.07*	–0.06	0.10**	0.09*	0.57**	1		
8 FI	9.67	4.61	−0.07**	0.02	−0.10**	0.10**	0.06	0.51**	0.55**	1	
9 Neuroticism	21.36	7.51	0.07*	–0.02	−0.09**	0.08*	0.03	0.66**	0.31**	0.44**	1

N = 814. AMHI, average monthly household income; ADSU, average daily smartphone usage time; NE, negative emotions; CP, cyberbullying perpetration; FI, family incivility. *p < 0.05, **p < 0.01.

### Testing for mediating effect of negative emotions

Table 2 showed that family incivility was positively related to negative emotions (β = 0.27, *p* < 0.001), and negative emotions was positively associated with cyberbullying perpetration (β = 0.20, *p* < 0.001). Finally, it was found that family incivility had an indirect effect on cyberbullying perpetration (β = 0.18). Bootstrapping results confirmed the significance of the indirect effect, with a 95% confident interval of [0.133, 0.327]. Therefore, H2 was supported.

**TABLE 2:** Testing the mediation effect of negative emotions on cyberbullying perpetration (*N* = 814).

Predictor (s)	Model 1: NE		Model 2: CP	
	β	*t*	β	*t*
Gender	–1.39	–2.02	–1.53	−4.59***
Age	0.61	5.51	–0.06	–1.14
FI	0.27	4.40***	0.18	7.98***
NE			0.2	11.40***
*R* ^2^	0.27		0.39	
*F*	97.60***		128.1***	

N = 814. NE, negative emotions; CP, cyberbullying perpetration; FI, family incivility. ***p < 0.001.

### Testing for moderated mediation

The results for H3 and H4 are reported in Table 3. Results demonstrated that the interaction of family incivility with neuroticism significantly predicted negative emotions (β = 0.04, *p* < 0.001) and cyberbullying perpetration (β = 0.03, *p* < 0.001).

**TABLE 3:** Testing the moderated mediation effect of neuroticism on cyberbullying perpetration.

Predictor (s)	Model 1: NE		Model 2: CP	
	β	*t*	β	*t*
Gender	–2.33	–4.25	–0.09	−3.39***
Age	0.61	6.97	–0.08	–1.43
FI	0.4	5.65***	0.33	7.91***
Neuroticism	0.84	21.36***	–0.09	–3.28
FI × Neuroticism	0.04	5.61***	0.03	8.83***
NE			0.22	10.51***
*R* ^2^	0.54		0.46	
*F*	189.27***		112.32***	

N = 814. NE, negative emotions; CP, cyberbullying perpetration; FI, family incivility. ***p < 0.001.

**FIGURE 2:**
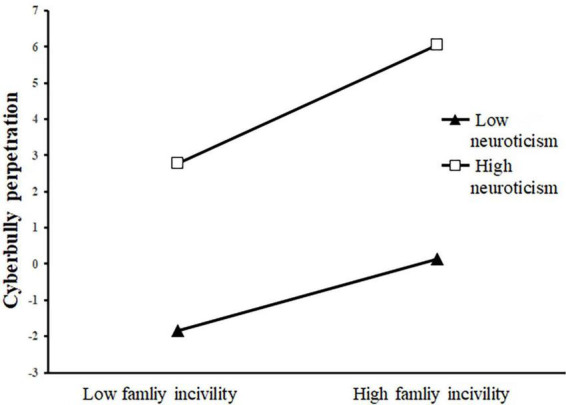
Interaction effect of family incivility and neuroticism on cyberbully perpetration. The black triangle represents low neuroticism and the white square represents high neuroticism.

**FIGURE 3:**
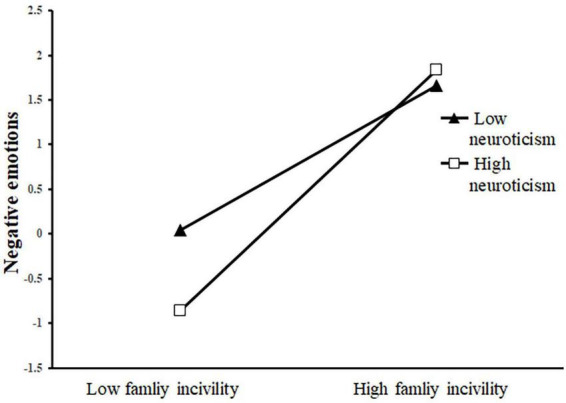
Interaction effect of family incivility and neuroticism on Negative emotions. The black triangle represents low neuroticism and the white square represents high neuroticism.

Next, we plotted simple slopes which predicted the relationship between family incivility and negative emotions as well as that between family incivility and cyberbullying perpetration, separately for high and low levels of neuroticism. As presented in Figure 2, the slope of the association between family incivility and negative emotions was relatively strong for participants with high levels of neuroticism (β_*high Neuroticism*_ = 7.44, *t* = 9.84, *p* < 0.001). When participants with low levels of neuroticism, the moderating association between family incivility and negative emotions was insignificant (*β_*low*_*_*Neuroticism*_ = −7.44, *t* = 1.34, *p* = 0.18). Additionally, as shown in Figure 3, the effect of family incivility on cyberbullying perpetration was stronger for participants with high levels of neuroticism (β_*high Neuroticism*_ = 7.44, *t* = 0.04, *p* < 0.001), whereas the moderating association between family incivility and cyberbullying perpetration was insignificant for participants with low levels of neuroticism (*β_*low*_*_*Neuroticism*_ = −7.44, *t* = 0.06, *p* = 0.148).

## Discussion

The current study aims at investigating how family incivility affects university students’ cyberbullying perpetration, as well as the role of negative emotions and neuroticism in the above relationship, which enriches previous research on family incivility and extends the frustration-aggression theory. Specifically, the results indicated that family incivility was positively related to cyberbullying perpetration among university students through negative emotions. The effects of family incivility on negative emotions and cyberbullying perpetration were stronger for university students with high levels of neuroticism. However, low levels of neuroticism hardly moderated the relationship between family incivility and negative emotions, as well as that between family incivility and cyberbullying perpetration, which is consistent with previous studies demonstrating that low levels of neuroticism hardly affected individual mental health problems and problematic behaviors (Kuang et al., 2020; Wang et al., 2021). Contrary to highly neurotic individuals, those at a low level were always feeling more relaxed and imperturbable (Mccrae and Costa, 1987) and therefore had less negative emotional experience of stressful life events, such as family incivility.

### Theoretical contributions

Our study contributed to the current literature from four aspects. Firstly, although abundant research has been conducted in the field of family incivility, most of them were focused on the effect of family incivility in the family work context (Bai et al., 2016; Wright et al., 2020; Ren et al., 2021). Little attention has been paid to the relationship between family incivility and cyberbullying perpetration among university students in the family school context. In the present study, we put family incivility into the theoretical framework of the frustration-aggression theory, finding that chronic frustration (i.e., family incivility) also influenced university students’ cyberbullying perpetration, supporting Hypothesis 1. That is consistent with the previous research, showing that stressful life events resulted in the person’s cyberbullying perpetration (Yudes et al., 2021). Gurr (1970) found that the repeated and chronic frustration can cause the outbreak of aggression at both individual and social levels. Along with a constant source of frustration, the victims experiencing more family incivility are more likely to perpetrate cyberbullying. Besides, the social status of the target is a potential moderator (Cohen, 1955), and retaliation is more likely to happen and increase in an anonymous environment (Rule et al., 1978), where the imbalance of power is eliminated to large extent, so that anyone, even weak individuals or from lower social class, can attack others online (Barlett and Gentile, 2012). Our study provides a new perspective for exploring the effects of family incivility and the intervention of cyberbullying in universities.

Secondly, we examined the mediating role of negative emotions and found that negative emotions played a mediating role between family incivility and cyberbullying perpetration, supporting Hypothesis 2. Previous studies have demonstrated that stress, anxiety, and depression were the most frequent mental illnesses among university students (Paudel et al., 2020), which were positively related to cyberbullying perpetration (Schodt et al., 2021). A prior study claimed that negative emotions, such as stress and anxiety, can lead to individuals’ impulsivity (Metcalfe and Mischel, 1999). The persons with high levels of impulsivity are more inclined to cyberbullying perpetration (Kowalski et al., 2014). However, previous research has paid little attention to the stressor, that is, family incivility. As a matter of fact, external stimuli, that is, stressors, can only produce general arousal, while the way an individual interprets his internal perception influences his perception of stressful life events and aggression much more (Berkowitz, 1978). Family incivility as a stressor can cause real disturbance or pressure, creating individual negative emotions that is an instigation for the person to perpetrate cyberbullying. Many studies have explored the impacts of family incivility on individual negative emotions and aggressive acts (Maria and Devi, 2020). Family incivility (e.g., familial indifference, exclusion, or privacy inquiry) can make individuals feel more psychological distress (Lim and Tai, 2014) and emotional consumption (Hassan et al., 2019), then experiencing more negative emotions further. With more family incivility for a long duration, individuals may fail to release their negative emotions timely and effectively, thus causing their cyberbullying perpetration. Therefore, through examining the relationship between family incivility and negative emotions, it is plausible that university students experiencing more family incivility will develop more negative emotions and be more likely to carry out aggressive acts, such as cyberbullying perpetration. Our study stresses the significance of university students’ mentality, associating their prior experiences with problem behaviors and offering a new angle to inspect the association between family interactions and aggressive acts online.

Thirdly, the results partially support the moderating role of neuroticism. Neuroticism reinforces the person’s stress responses and the person with neuroticism is more vulnerable to stress (Suls, 2001). Neurotics are more likely to experience pain and negative emotions (Mccrae and Costa, 1987), which prepares them for perceiving threats (Schneider, 2004). In face of stressful life events, highly neurotic individuals are easily affected by negative cognition and feel a lack of strategic resources to stressors, and accordingly they may regard stressful life events as a threat more easily (Gallagher, 1990). A two-year longitudinal study showed that a threat appraisal to stressful life events resulted in the person’s negative emotions, such as anxiety and depression, and aggressive behaviors (Taylor et al., 2013). As shown in the results, high levels of neuroticism strengthened the correlation between family incivility and negative emotions, as well as the correlation between family incivility and cyberbullying perpetration. Hypotheses 3 and 4 were partially supported. However, our findings are consistent with previous studies showing that university students with high levels of neuroticism are more susceptible to stressors, leading to negative emotions and cyberbullying perpetration, while those with low levels of neuroticism are less likely to have such problems (Miceli et al., 2021). Neuroticism influences the person’s ability of emotional control. Individuals with different levels of neuroticism are different from selective attention, cognitive appraisals, and coping strategies (Mccrae and Costa, 1987). In face of stressful life events, highly neurotic individuals with poor emotional regulation ability are more sensitive to negative information and possibly experience a higher level of negative emotions, making their emotional interpretation problematic (Horner, 1996). In addition, individuals with high levels of neuroticism are more inclined to adopt negative cognitive appraisal, considering family incivility (i.e., the stressor) threatening (Schneider, 2004), eventually leading individuals to adopt negative coping strategies (e.g., cyberbullying perpetration), to alleviate their negative emotions. A recent biological experiment also showed that high neuroticism can increase individuals’ reactivity of limbic system and decrease their tolerance to stressors or aversive stimuli, so that highly neurotic individuals are always disturbed by negative emotions and adopt negative coping strategies (Magal et al., 2021). Our study reflects individual differences among victims suffering from family incivility, and neuroticism plays an important role in this moderated mediation model.

Finally, this study enriches the applicability of the frustration-aggression theory among contemporary university students. It provides empirical support for this theory and explores the interactive interface between online and offline environments. The family incivility is a low-intensity and inconspicuous stressful life events (Lim and Tai, 2014). Therefore, it is always difficult to find that family incivility is an incentive to university students’ negative emotions and cyberbullying perpetration. This study found that family incivility was positively associated cyberbullying perpetration directly or through the mediator of negative emotions, indicating that chronic negative experiences in real life can also cause online aggressive acts. This study provides a new perspective for the frustration-aggression theory in interpreting the influence of frustration strength on aggressive behavior. In addition, personality, as a relatively stable individual trait, can chronically affect individual cognition and behavioral style (Back et al., 2009). Combined with the Five – Factor Model of personality, this paper discusses the mechanism of neuroticism in the frustration-aggression theory, which also provides an empirical test for the mechanism of personality traits in stress research.

### Practical implications

This research also has some practical implications. As the findings of this study, there was a positive correlation between family incivility, negative emotions, and cyberbullying perpetration, suggesting that parents should avoid negative family interactions,such as neglect, rejection and probing into privacy, and establish a respectful, harmonious and intimate family relationship to restrain individual negative emotions and cyberbullying perpetration.

Moreover, university students’ mental health is closely related to their growing experience. The research reveals that the lasting influence of negative family interaction on university students is hardly weakened even if they have left their families away to live in a new environment. Therefore, psychological education should be united with students’ families, which is beneficial to prevent university students’ negative emotions from the source and intervene the vicious circle of cyberbullying in university efficiently.

Finally, the findings suggest that neuroticism plays an important role in how individuals interact with stressful life events. Highly neurotic individuals are more vulnerable to pressure events and prone to cyberbullying. Therefore, for university students with high levels of neuroticism, they should learn to manage their emotions and maintain emotional stability, to alleviate the negative emotions caused by family incivility.

### Limitations and future research

The current study still has several limitations. First, this study is a cross-sectional study rather than a longitudinal one, so that we can hardly evaluate the causal relationship between various variables. As reported by Acker and Pitchford (2014), family intimacy among university students was positively related to their peer intimacy and life satisfaction that are the two main factors for students’ negative emotions and aggressive behaviors (Huang et al., 2021; Yokotani and Takano, 2021). In our study, nearly half of the participants were freshmen who may have a strong relationship with their family, for which most of them would be more affected by family incivility. However, precious studies found that the family influence on university students was dynamic in terms of their age and grade (Lopez, 1995). Therefore, further studies are needed to extend the relationship between family incivility and cyberbullying perpetration longitudinally.

Secondly, to preliminarily reflect the impact of family incivility on the mental health of Chinese university students, this study takes negative emotions (namely stress, anxiety, and depression) as an overall intermediary variable, but to some extent fails to reflect the stronger impact of family incivility on negative emotions among the three dimensions. However, anxiety and stress are phenomenologically different (Henry and Crawford, 2011). Future studies could be extended the mediating role of stress, anxiety, and depression prospectively, to gain full understanding on how these three psychologically distinct negative emotions play a role in the relationship between family incivility and cyberbullying perpetration.

Finally, to improve the theoretical construction and practical application of incivility in frustration-aggression model (Berkowitz, 2003), the influence of family incivility on daily aggressive behavior of university students can be further explored in the future. Meanwhile, some protective factors on the relationship of family incivility and bullying can be explored, such as peer support, individual positive traits, rumination (Naylor and Cowie, 1999; Muris et al., 2005).

## Conclusion

We investigated the correlations between family incivility and cyberbullying perpetration among Chinese university students and examined the mediated moderation model of negative emotions and neuroticism. Overall, family incivility was positively correlated with negative emotions and cyberbullying perpetration among university students. Negative emotion played a mediating role in the influence of family incivility on cyberbullying perpetration. Neuroticism can regulate the impact of family incivility on negative emotions and cyberbullying perpetration prospectively. High levels of neuroticism can increase the impact of family incivility on cyberbullying perpetration and on negative emotions, while low levels of neuroticism had no such effect on the relationships. This study provides an insight for exploring how family incivility affects university students’ negative emotions and aggression. It also constructs a theoretical model for how family incivility affects the development of university students.

## Data availability statement

The raw data supporting the conclusions of this article will be made available by the authors, without undue reservation.

## Ethics statement

The studies involving human participants were reviewed and approved by the Ethics Committee of Faculty of Psychology, Ningbo University. Written informed consent to participate in this study was provided by the participants and the participants’ legal guardian/next of kin. Written informed consent was obtained from the individual(s), and minor(s)’ legal guardian/next of kin, for the publication of any potentially identifiable images or data included in this article.

## Author contributions

JG, JX, and FL designed the work. JM and JW analyzed the data results. JX drafted the manuscript. JG, FL, and LW revised the manuscript. All authors contributed to the article and approved the submitted version.
